# Dynamic behavior of double-cube multi-bubble system driven by square waves

**DOI:** 10.1016/j.ultsonch.2026.107955

**Published:** 2026-07-14

**Authors:** Wurihan Bao, De-Xin Wang, Haiying Han

**Affiliations:** College of Physics and Electronics, Inner Mongolia Minzu University, Tongliao 028043, China

**Keywords:** Multi-bubble dynamics, Double-cube structure, Square wave driving, Secondary Bjerknes force

## Abstract

This study investigates the radial dynamics of a hierarchically arranged 17-bubble system consisting of eight outer-cube bubbles, eight inner-cube bubbles, and one central bubble under finite-bandwidth square-wave excitation. The bubble positions are defined by a Cartesian coordinate model, from which the complete pairwise distance matrix is incorporated to evaluate collapse temperature, rebound, and wall velocity without relying solely on a polytropic approximation, with coupling terms fully introduced into a compressible multi-bubble equation. Non-equilibrium acoustic pressure, frequency, geometric scale ratio, bubble-size ratio, viscosity, surface tension, evaporation/condensation and interfacial thermal diffusion are characterized via dimensionless parameters to quantify coupling effects. The results identify normal expansion, complete suppression, and delayed expansion regimes of the central bubble and show that these regimes are governed by the competition between the externally imposed tensile pressure and the radiation pressure generated by the surrounding bubbles. Square-wave and sinusoidal forcing are compared under both equal-peak-pressure and equal-RMS-pressure conditions. The maximum wall Mach number and rebound amplitude are reported to delimit the validity of the spherical Keller–Miksis-based formulation. The calculated collapse temperature is interpreted as a thermodynamic severity metric rather than a direct measure of oxidant yield, because sonochemical radical production may decrease above an optimum bubble temperature. The model clarifies the parameter ranges in which a fixed-centre radial description is appropriate and the ranges in which migration- and deformation-resolving models are required.

## 1 Introduction

When ultrasonic waves propagate in a liquid medium, the density state of liquid molecules changes periodically. After the acoustic pressure reaches a specific threshold, tiny gas nuclei in the liquid will expand to form observable bubbles, a process generally referred to as acoustic cavitation [1], [2], [3]. The rapid growth and collapse of cavitation bubbles can generate shock waves, high local temperatures and intense fluid motion, providing the physical basis for sonochemistry, cleaning, emulsification and related ultrasonic processes [2], [3], [4], [5]. Acoustic cavitation technology has shown extensive application value in various industrial fields, including homogenization in emulsification processes, ultrasonic cleaning of precision parts, catalytic enhancement of chemical reactions, wastewater treatment and biomedicine. Actual application scenarios often involve bubble cluster systems composed of a large number of cavitation bubbles, rather than single isolated bubbles, making the interaction mechanism between bubbles a key link in understanding and optimizing cavitation effects. The complexity of bubble interaction is also the main factor restricting the industrialization of high-efficiency acoustic cavitation technology.

The radial dynamics of an isolated spherical bubble are commonly described by Rayleigh–Plesset-type equations. For violent oscillations in which liquid compressibility becomes important, the Keller–Miksis equation and the first- and second-order compressible-liquid formulations of Prosperetti and Lezzi provide widely used theoretical foundations [6], [7], [8]. Thermal conduction and mass transfer can substantially modify the pressure and temperature reached during collapse, meaning that a fixed polytropic exponent is generally insufficient for quantitatively predicting strong cavitation events [9].

The theoretical description of bubble–bubble interaction is commonly developed by coupling the radial equations of individual bubbles through the acoustic pressure radiated by their neighbours. For an isolated spherical bubble undergoing large-amplitude oscillation, liquid compressibility can be incorporated through the Keller–Miksis equation and related first- and second-order compressible-liquid formulations [6], [7], [8]. When two or more bubbles are present, the pressure emitted by one bubble modifies the instantaneous forcing experienced by the others, leading to coupled changes in expansion amplitude, collapse timing and acoustic emission [10], [11]. In addition to radial coupling, differences in oscillation amplitude and phase generate a time-averaged interaction known as the secondary Bjerknes force, which may be attractive or repulsive depending on the operating conditions [12], [13]. More general formulations have demonstrated that translational motion, strong nonlinear coupling and bubble deformation can further modify the interaction dynamics [14], [15], [16], [17], [18].

Research has subsequently progressed from isolated bubble pairs to multi-bubble clouds and structured bubble clusters. Chahine and Duraiswami developed a numerical description of dynamical interactions in multi-bubble clouds, demonstrating that the response of each bubble is influenced by the collective pressure field generated by the surrounding population [19]. Particle-based and cluster-scale studies further showed that acoustic interactions contribute to the spatial organization, collective oscillation and energy redistribution of cavitation bubbles [20], [21], [22]. At the level of a finite number of interacting bubbles, numerical studies have demonstrated that the radiated pressure from neighbouring bubbles can either attenuate or enhance radial oscillation. The effect is particularly pronounced for a small bubble located near one or more larger bubbles and depends on the equilibrium radii, separation distances, driving frequency, acoustic-pressure amplitude and number of interacting bubbles [10], [11]. These findings provide the physical basis for examining whether a hierarchically arranged double-cube configuration can produce suppression or delayed response of its central bubble.

Controlled non-sinusoidal waveforms alter the duration of the tensile stage and introduce harmonic components that are absent from a single-frequency sinusoid [23], [24]. Previous numerical and experimental studies show that these waveform changes can modify bubble expansion, mass transfer and sonochemical activity [23], [24], [25]. However, the interpretation depends on whether peak pressure, RMS pressure, acoustic power or total input energy is held constant.

Collapse temperature is used here as an indicator of thermodynamic severity rather than a direct measure of oxidant production. Non-equilibrium vapour transport can limit the temperature reached during collapse, while chemical models and experiments indicate that oxidant production has an optimum temperature range rather than increasing monotonically with temperature [26], [27], [28], [29], [30], [31].

The mathematical descriptions in existing bubble dynamic models struggle to fully reflect the complex spatial distribution characteristics of bubble arrays in actual cavitation fields. As the most basic bubble interaction unit, the double-bubble system has been widely studied with abundant research outcomes. Inspired by the above research progress, this paper constructs a hierarchically nested multi-bubble spatial model with perfect cubic symmetry. The model consists of eight outer-cube vertex bubbles, eight inner-cube vertex bubbles, and one central bubble positioned at the geometric origin of the whole configuration, forming a fully coupled three-dimensional system containing 17 spherical cavitation bubbles. The centre-to-vertex characteristic size of the outer cube is defined as D, which represents the reference separation scale between outer-layer bubbles; the centre-to-vertex characteristic size of the inner cube is denoted as d, satisfying the constraint d≤D to realize the nested layout of inner and outer cube frameworks. This double-cube geometric configuration retains a high level of spatial symmetry while forming an obvious inward hierarchical structure. The central bubble simultaneously receives acoustic radiation excitation from all 16 surrounding bubbles distributed in the inner and outer layers, such that the composite acoustic pressure field acting on the central bubble is far more complex than that of simple linear bubble chains or single-layer spherical bubble clusters.

The centre distance between every pair of bubbles is generated from an explicit Cartesian-coordinate model. This replaces the ambiguous ${\mathrm{hh}}^{2}+{\mathrm{hh}}^{2}$ notation in the initial manuscript and ensures that every distance appearing in the 17-bubble interaction equation is uniquely defined.

The coupling characteristics in complex bubble arrays. The double-cube multi-bubble system possesses a high degree of geometric symmetry and hierarchical structure. The nested configuration of the outer cube and inner cube forms a unique spatial field distribution. The central bubble is situated in the combined acoustic field of multiple surrounding bubbles, and its dynamic behavior is fundamentally different from simple linear or spherical bubble clusters. The coupling effects of multi-bubble systems under square wave driving have not yet been systematically studied.

This paper establishes a double-cube multi-bubble model containing 8 bubbles in the outer cube, 8 bubbles in the inner cube, and 1 central bubble. It derives the coupled dynamic equations for the 17 bubbles and numerically solves the system under square wave excitation. The focus is on studying the dynamic characteristics, secondary Bjerknes force distribution laws, and cavitation effects of the central bubble under complex geometric fields and multi-bubble coupling, providing a theoretical basis for the design and optimization of acoustic cavitation bubble arrays.

## 2 Double-cube multi-bubble model and governing equations

Existing multi-bubble dynamics research mostly focuses on regular geometric configurations such as linear and spherical shapes. Although these models simplify calculation efficiency.

Although existing research has achieved rich results in double-bubble, three-bubble, five-bubble, and uniform spherical bubble clusters, the geometric configurations of these models are relatively simple and difficult to fully reflect spherical bubble clusters.

### 2.1 Cartesian coordinates and complete distance matrix

Let D and d denote the centre-to-vertex radii of the outer and inner cubes, respectively. The eight outer vertices are $r_{o}=\left(D/\sqrt{3}\right)\left(s_{x},s_{y},s_{z}\right)$, $r_{i}=\left(d/\sqrt{3}\right)\left(s_{x},s_{y},s_{z}\right)$, $s_{x},s_{y},s_{z}$ independently ±1. The central bubble is located at $r_{c}=\left(0,0,0\right)$. For any bubbles i and j, the separation used in the coupling equation is

(1) $$D_{ij}={∥r_{i}-r_{j}∥}_{2}.$$

This coordinate definition automatically produces all same-layer and cross-layer distances. Within one cube of centre-to-vertex radius a, the edge, face-diagonal, and body diagonal lengths are $2a/\sqrt{3}$, $2a\sqrt{2/3}$, $2a\sqrt{3}$ respectively. For an outer-inner pair with sign vectors s,q

(2) $$D_{oi}\left(s,q\right)={\left[D^{2}+d^{2}-\left(2Dd/3\right)\left(s_{x}q_{x}+s_{y}q_{y}+s_{z}q_{z}\right)\right]}^{1/2}.$$

The centre-to-outer and centre-to-inner distances are D and d, respectively. The distance matrix is 17×17 zero diagonal entries and 136 unique off-diagonal separations. The full coupled Keller–Miksis system uses the corresponding $D_{ij}$ for every interacting pair; no single representative distance value is substituted for the full geometry. Thus, the difference from a generic multi-bubble model lies not in the algebraic form of the coupling term but in the explicitly generated hierarchical distance matrix and in the three bubble-size classes.

The outer, inner, and central coordinates, together with D, d, and representative pairwise distances, should be read directly from the coordinate construction above.

Expressed as 2, and its body diagonal length is $2\sqrt{3}$. The corresponding side length and body diagonal of the inner cube are 2 and $2\sqrt{3}$, respectively. To facilitate subsequent equation derivation, a characteristic length $h=2\sqrt{3}$ is introduced as the small side length of the outer cube. Accordingly, the large side length of the inner-outer composite cube can be defined as $hh=2\sqrt{3}+D$. Based on D and d, the complexity of the coupling relationship increases exponentially.

The effective pressure $p_{s,i}$ on the bubble wall is formed by the superposition of multiple components:

(3) $$p_{s,i}=p_{B,i}-p_{0}-p_{d}\left(t\right)-2\sigma /R_{i}-4\mu {\overset{˙}{R}}_{i}/R_{i}.$$

The inner cube follows the same geometric laws, with adjacent and diagonal bubble spacings being $2\sqrt{2/3}d$ respectively. For the distance between outer and inner bubbles, the oblique relationship in spatial geometry needs to be considered. The distance difference between vertices in the same direction satisfies $D-d=2\sqrt{2/3}$, while the distance between vertices in different directions needs to be calculated through spatial vector relationships. Specifically, this definition, the distance between adjacent vertex bubbles in the outer cube is:

(4) $$\sqrt{hh^{2}+hh^{2}}=2\sqrt{2}\;h$$

The distance between diagonal vertices is: the distance from the central bubble to the 8 vertex bubbles of the outer layer is $\sqrt{3}D$, and the distance to the 8 vertex bubbles of the inner layer is $\sqrt{3}d$. This equidistant distribution reflects the high symmetry of the system, which is of great significance for simplifying subsequent analysis.

When describing the radial vibration of mutually coupled multi-bubble systems in compressible liquids, the modified Keller–Miksis equation considering liquid compressibility and acoustic coupling between bubbles is adopted as the governing equation. For the ith bubble in the system (i=1,2,…,17), its radial motion satisfies the coupled Keller–Miksis formulation:

(5) $$\left(1-\frac{{\overset{˙}{R}}_{i}}{c}\right)R_{i}{\overset{¨}{R}}_{i}+\frac{3}{2}\left(1-\frac{{\overset{˙}{R}}_{i}}{3c}\right){\overset{˙}{R}}_{i}^{2}=\frac{1}{\rho }\left(1+\frac{{\overset{˙}{R}}_{i}}{c}\right)p_{s,i}+\frac{R_{i}}{\rho c}\frac{dp_{s,i}}{dt}-\underset{j\neq i}{\sum }\frac{\rho }{D_{ij}}\frac{d}{dt}\left(R_{j}^{2}{\overset{˙}{R}}_{j}\right),$$

The isolated-bubble part of the governing equation follows the first-order compressible formulation of Keller and Miksis [6], which belongs to the family of weakly compressible spherical-bubble equations discussed by Prosperetti and Lezzi [7], [8]. The mutual-interaction term represents the pressure emitted by neighbouring spherical bubbles and follows the coupled-bubble approximation used in previous multi-bubble studies [10], [11], [19].

$R_{i0}$ is the initial equilibrium radius of the ith bubble, κ is the polytropic index of the gas (taken as κ=1.4 for air), and $a_{v}$ is the van der Waals hard-core radius ($a_{v}=0.85\;\mu \text{m}$ for air bubbles). The selected gas equation of state can significantly influence the predicted minimum radius, collapse pressure and temperature. Recent comparisons show increasing differences between van der Waals and more advanced equations of state as collapse intensity increases [32]. This equation of state is closer to the thermodynamic behavior of real bubbles than the ideal gas law, especially when intermolecular forces cannot be ignored during violent bubble collapse.

The external driving acoustic pressure $p_{d}\left(t\right)$ adopts a square wave form, which can be expressed through Fourier series expansion as:

(6) $$p_{d}\left(t\right)=\overset{N}{\underset{n=1}{\sum }}\frac{4P_{a}}{\pi \left(2n-1\right)}{\left(-1\right)}^{n+1}\cos\left[\left(2n-1\right)\omega t\right].$$

Where $P_{a}$ is the square wave amplitude, N is series truncation terms; a sufficiently large N approximates an ideal square wave, but finite truncation is adopted for numerical calculation.

The finite-bandwidth square wave differs from a sinusoid through its longer nearly constant tensile interval and its odd-harmonic content. To avoid attributing an energy-input difference entirely to waveform shape, the revised study distinguishes equal-peak-pressure and equal-RMS-pressure comparisons. Under equal peak pressure, a square wave has a larger RMS pressure than a sinusoid; therefore, stronger expansion in that comparison partly reflects the larger cycle-averaged forcing. The equal-RMS comparison is used to isolate the effect of waveform shape and spectral content.

The secondary Bjerknes force between bubbles originates from the phase and amplitude differences in the volume pulsation of adjacent bubbles. For the ith bubble, the secondary Bjerknes force it receives from the jth bubble is:

(7) $$F_{Bij}=-\frac{\rho }{4\pi D_{ij}^{2}}\langle {\overset{˙}{V}}_{i}{\overset{˙}{V}}_{j}\rangle e_{ij},$$

This expression corresponds to the leading-order time-averaged interaction between radially pulsating spherical bubbles [12], [13]. It does not include translational, deformational or convective corrections, which can alter both the magnitude and sign of the interaction force under strong excitation [14], [15], [16], [17], [18]. $V_{i}$ and $V_{j}$ are the instantaneous volumes of the two bubbles, respectively. The angle brackets indicate time averaging, and $e_{ij}$ is the radial unit vector. When $\langle {\overset{˙}{V}}_{i}{\overset{˙}{V}}_{j}\rangle >0$, it manifests as a repulsive force; when $\langle {\overset{˙}{V}}_{i}{\overset{˙}{V}}_{j}\rangle <0$, it is an attractive force. In the double-cube system of this paper, the central bubble is simultaneously subjected to the secondary Bjerknes forces from 16 surrounding bubbles, and the total force is the vector sum of the component forces. Due to the cubic symmetry of the system, the radial component forces of the outer 8 bubbles on the central bubble and the radial component forces of the inner 8 bubbles will partially cancel each other out under ideal symmetrical conditions. However, if there are slight differences in bubble size or phase, symmetry breaking will cause the central bubble to be subjected to a net lateral force, thereby inducing a trend of movement away from the geometric center.

During model establishment, the liquid is treated as homogeneous and initially quiescent, and the bubble centres are fixed so that the present calculation resolves radial oscillation only. Spherical symmetry is retained while the maximum wall Mach number and the ratio $\left(R_{i,\text{max}}+R_{j,\text{max}}\right)/D_{ij}$ are monitored to identify conditions under which deformation, overlap, or migration may invalidate the model. Unlike the initial version, the revised thermodynamic closure includes non-equilibrium evaporation/condensation of water vapor and interfacial heat conduction. Chemical reactions are not solved explicitly; therefore, the calculated temperature is not used as a direct proxy for oxidant yield. The model assumes an unbounded liquid domain and neglects walls. At t=0, $R_{i}=R_{i0}$ and $dR_{i}/dt=0$ for all bubbles.

The Keller–Miksis-based formulation is selected because the primary objective is the coupled radial response of a symmetric, fixed-centre array under periodic acoustic forcing, for which first-order liquid compressibility and radiation coupling are required at moderate computational cost. Migration-resolving Zhang-type equations are more general when translation, dynamic drag, and asymmetric deformation are central outputs. Recent work by Xu et al. extends that framework across dynamic Reynolds numbers and includes deformation corrections [33]. The present model is therefore not claimed to replace the Zhang framework: it defines a radial baseline. Whenever the computed symmetry-breaking force, wall Mach number, or geometric proximity criterion becomes large, migration- and deformation-resolving calculations are required.

### 2.2 Non-equilibrium vapor transport and interfacial heat transfer

The spatially averaged gas-vapor energy equation is written as

(8) $$\frac{dU}{dt}=-p_{B}\frac{dV}{dt}+4\pi R^{2}q_{w}+h_{v}\frac{dm_{v}}{dt},$$

where $q_{w}=-k_{l}\left(\partial T/\partial r\right){|}_{R}$ is the conductive heat flux into the bubble and $h_{v}$ is the vapor enthalpy contribution. A boundary-layer approximation is used for heat transfer, $q_{w}\approx -k_{l}\left(T_{B}-T_{\infty }\right)/\delta_{T}$, with $\delta_{T}=\min\left[R,\sqrt{\alpha_{l}/\omega_{\text{eff}}}\right]$. The gas temperature $T_{B}$, vapor content, and pressure are therefore evolved consistently rather than inferred solely from a fixed polytropic exponent. The energy closure follows the established treatment of heat transfer in oscillating gas bubbles [9], while the inclusion of non-equilibrium evaporation and condensation is motivated by previous theoretical studies demonstrating the importance of phase change during rapid collapse [26], [27], [28], [29].

The revised calculation separates non-condensable gas and water vapor. The total internal pressure is $p_{B}=p_{g}+p_{v}$, and the vapor mass changes through a non-equilibrium Hertz–Knudsen interfacial flux:

(9) $$\frac{dm_{v}}{dt}=4\pi R^{2}J_{v},\;J_{v}=\alpha_{m}\frac{p_{\text{sat}}\left(T_{s}\right)-p_{v}}{\sqrt{2\pi R_{v}T_{s}}}.$$

Here $\alpha_{m}$ is the accommodation coefficient, $p_{\text{sat}}$ satisfies the saturation vapor pressure at the interface temperature $T_{s}$, and $R_{v}$ is the specific gas constant of water vapor. Positive $J_{v}$ denotes evaporation and negative $J_{v}$ denotes condensation. The accommodation coefficient and interfacial temperature treatment must be specified because phase-change predictions can be sensitive to the adopted interfacial closure [26], [27].

### 2.3 Finite-bandwidth forcing, wall mach number, and rebound measures

The wall pressure in Eq. (5) is consequently revised to $p_{s,i}=p_{B,i}-p_{0}-p_{d}\left(t\right)-2\sigma /R_{i}-4\mu {\overset{˙}{R}}_{i}/R_{i}$. The original polytropic relation is retained only as a comparison closure in the model-verification subsection.

A physically finite square wave is represented by N odd harmonics rather than an unresolvable ideal discontinuity. Convergence is checked by increasing N until $R_{\text{max}}$, the first-collapse time, the rebound ratio, and the maximum wall Mach number change by less than 1%. The revised reference calculation uses N=25; the statement $n=10^{9}$ in the initial version is removed because harmonics at that level cannot be resolved by the numerical time step.

The maximum wall Mach number and rebound ratio are defined as $M_{w,\text{max}}=\max|\overset{˙}{R}|/c_{w}$ and $\chi_{\text{reb}}=R_{\text{reb,max}}/R_{\text{pre-collapse,max}}$, respectively. The local liquid sound speed $c_{w}$ is evaluated at the bubble wall. Results with $M_{w,\text{max}}$ approaching unity, or with neighboring expanded bubbles satisfying $\left(R_{i}+R_{j}\right)/D_{ij}\geq 0.5$, are treated as outside the quantitatively reliable range of the fixed-centre spherical model.

### 2.4 Choice of radial model and relation to migration-resolving equations

Spherical radial models are computationally efficient but do not resolve jetting, translation or shape deformation. Non-spherical boundary-integral and direct numerical simulations demonstrate that these effects become important during asymmetric or closely coupled collapse [34], [35]. More general theoretical models have therefore been developed to couple compressibility, phase transition, migration and deformation [33], [36]. Recent DNS comparisons also show that coupled spherical-bubble models can overpredict internal pressure when jetting occurs [37].

## 3 Numerical calculation methods and parameter settings

### 3.1 Numerical consistency checks and comparison with established coupled-bubble behaviour

For the double-cube multi-bubble coupled dynamic equation system established above, this paper uses the classic fourth-order Runge–Kutta algorithm for numerical integration. This algorithm is widely used in the field of bubble dynamics due to its good stability and calculation accuracy. For a system containing 17 bubbles, the motion of each bubble is described by a second-order ordinary differential equation. By introducing intermediate variables $u_{i}={\overset{˙}{R}}_{i}$, the original equation system can be transformed into an initial value problem of 34 first-order ordinary differential equations. Specifically, let the state $x=\left[R_{1},u_{1},R_{2},u_{2},\ldots ,R_{17},u_{17}\right]$, then the system dx/dt=f(x,t).

The selection of the time step for numerical integration needs to comprehensively consider computational efficiency and accuracy requirements. The radius change during the bubble collapse stage is extremely drastic. If the step size is too large, it will lead to numerical oscillation or even divergence; while in the slow expansion stage of the bubble, a step size that is too small will cause a waste of computational resources. After multiple trial calculations and convergence tests, this paper selects a $\Delta t=1\times 10^{-9}\;\text{s}$. This step size can accurately capture the rapid dynamic process at the moment of bubble collapse and ensure the numerical stability of longterm integration. To verify the reliability of the numerical step, recalculation under typical working conditions with $\Delta t=5\times 10^{-10}\;\text{s}$. The results showed that the relative errors of the maximum bubble radius and the collapse moment were both less than 0.5%, indicating that the selected step size meets the accuracy requirements.

Special attention needs to be paid to the calculation of the coupling term summation acting on the ith bubble, and its time derivative needs to be obtained through numerical differentiation. This term involves the acoustic effect of other 16 bubbles, which has second-order accuracy and this paper adopts a central difference scheme to calculate the derivative term, matching the Runge–Kutta algorithm. For the initial moment, due to the lack of information at the −Δt time step, a forward difference scheme is used to start the calculation. The entire integration process lasts for at least 5 acoustic wave cycles to ensure that the system reaches steady-state oscillation or captures the complete dynamic behavior of the bubbles.

**Fig. 1:**
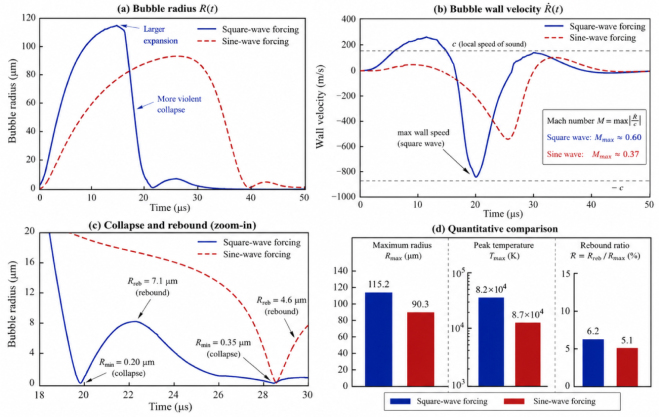
**Single-bubble dynamics under square-wave and sinusoidal forcing (same peak pressure).** (a) Bubble radius R(t). Square-wave forcing yields larger expansion and more violent collapse. (b) Bubble wall velocity $\overset{˙}{R}\left(t\right)$. The maximum inward wall speed under square-wave forcing is higher but remains bounded by the local speed of sound near the bubble wall (dashed lines); corresponding Mach numbers are listed. (c) Zoom-in of the collapse and rebound around the first collapse, showing a clear post-collapse rebound for both forcings, with a larger rebound radius for square-wave forcing. (d) Quantitative comparison of three key metrics: maximum radius $R_{\max}$, peak temperature $T_{\max}$, and rebound ratio $R=R_{\mathrm{reb}}/R_{\max}$. Square-wave forcing outperforms sinusoidal forcing in all metrics.

The single-bubble limit was obtained by setting all inter-bubble coupling terms to zero. Fig. 1 shows the resulting radius, wall velocity and rebound histories, which exhibit the established characteristics of a strongly driven Keller–Miksis bubble, including rapid tensile-stage expansion, violent collapse and a damped rebound [6], [7], [8]. The waveform-dependent differences are also qualitatively consistent with previous numerical studies of cavitation bubbles subjected to controlled non-sinusoidal excitation [23], [24]. This comparison is used as a qualitative model check; quantitative convergence is evaluated independently by reducing the integration time step and increasing the number of retained Fourier harmonics.

**Fig. 2:**
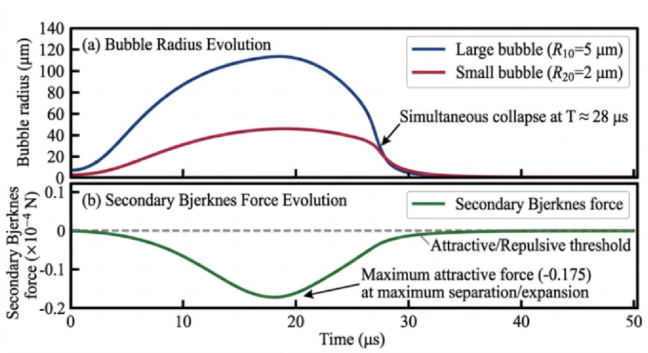
Dynamic characteristics of a double-bubble system.

Figure 2 gives the radius evolution and secondary Bjerknes force changes of a double-bubble system under square wave driving. The double-bubble calculation provides a numerical consistency check for the implementation of the acoustic-coupling term. Under the selected conditions, the larger bubble undergoes a substantially greater expansion than the smaller bubble, while the pressure radiated by the larger bubble attenuates the expansion of its neighbour. This qualitative behaviour is consistent with established coupled-bubble calculations showing that bubble–bubble interaction can strongly suppress the radial response of a smaller bubble while exerting a comparatively weaker influence on a larger one [10], [11]. Because the published studies employ different initial radii, separation distances, acoustic pressures and thermodynamic closures, no pointwise percentage error is assigned here. Numerical accuracy is instead assessed through the time-step and harmonic-truncation convergence tests described in Section 3.1.

### 3.2 Physical parameter settings

The physical parameters involved in the numerical simulation are divided into three categories: liquid physical properties, bubble geometric parameters, and acoustic field driving parameters. Pure water at room temperature and pressure is selected as the liquid medium, and its main physical parameters are shown in Table 1. Liquid density $\rho =998.2\;{\text{kg/m}}^{3}$, speed of sound c=1481m/s; these two parameters directly affect the compressibility term in the Keller–Miksis equation. Dynamic viscosity $\mu =1.0\times 10^{-3}\;\text{Pa}\cdot \text{s}$ determines the viscous dissipation intensity, and surface tension coefficient $\sigma =7.275\times 10^{-2}\;\text{N/m}$ reflects the constraint effect of surface energy on the bubble wall. The ambient static pressure is taken as $p_{0}=1.013\times 10^{5}\;\text{Pa}$. Bubbles are filled with air, the polytropic index κ=1.4, and the relationship between the van der Waals hard-core radius and the bubble equilibrium radius is $a_{v}=0.85\;\mu \text{m}$.

To examine the sensitivity of the numerical solution to the driving waveform, additional calculations were performed using sinusoidal and finite-bandwidth square-wave excitation. The comparison is not used as an independent validation of the solver because the two waveforms contain different harmonic components and may deliver different cycle-averaged energy. Instead, it provides a controlled assessment of how the prescribed pressure history affects the computed radial response [23], [24].

The driving acoustic field adopts a square wave form, square wave acoustic pressure amplitude is set to $1.3\times 10^{5}\;\text{Pa}$, slightly higher than the ambient pressure, which can drive the bubbles to produce obvious nonlinear oscillations. To explore the influence of driving intensity, $P_{a}$ is adjusted in the range of $1.0\times 10^{5}\;\text{Pa}$ to $1.5\times 10^{5}\;\text{Pa}$. The reference frequency is selected as f=20kHz, which is at the lower limit of the common ultrasonic cavitation range and is conducive to full bubble expansion. In the study of frequency influence laws, f varies in the range of 20 kHz and 35 kHz, with a step of 5 kHz.

**Fig. 3:**
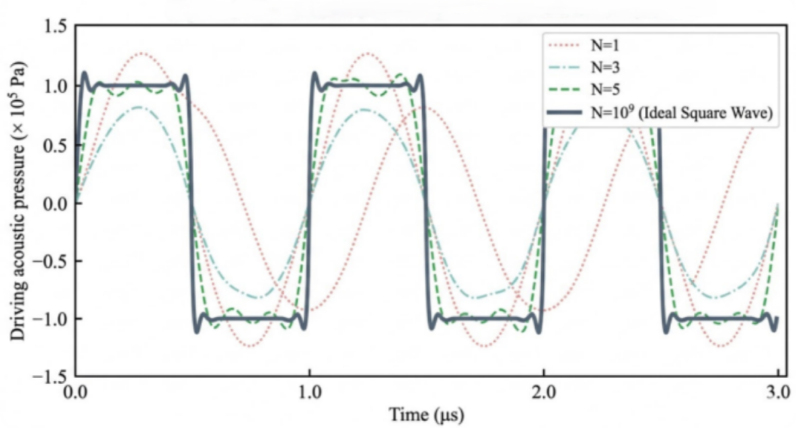
Square wave driving acoustic pressure waveforms under different numbers of truncation terms.

The number of truncation terms for the Fourier series expansion is analyzed in Figure 3. Ideal infinite truncation produces near-perfect square waveforms, while finite truncation introduces minor Gibbs oscillations at pressure jumps. Convergence testing confirms N=25 is sufficient for stable results.

The geometric parameter settings of the double-cube system need to comprehensively consider physical rationality and computational feasibility. The initial radius of the 8 vertex bubbles of the outer cube is uniformly set to $R_{o0}=5\;\mu \text{m}$, which corresponds to the larger bubbles observed in typical ultrasonic cavitation experiments. The initial radius of the 8 vertex bubbles of the inner cube is taken as $R_{i0}=2\;\mu \text{m}$, representing smaller cavitation nuclei. The initial radius of the 17th bubble located at the geometric center of the system is set to $R_{c0}=3\;\mu \text{m}$, between the sizes of the inner and outer bubbles. The characteristic dimension of the outer cube varies in the range of 100μm to 200μm, with the reference condition taken as D=150μm; the characteristic dimension of the inner cube is adjusted accordingly in the range of 50μm to 100μm, with the reference condition taken as d=75μm, making the inner/outer size ratio D/d=2.0. Such parameter configuration ensures that the bubble spacing is much larger than the bubble radius ($D_{ij}\gg 10R$), meeting the applicable conditions of the point source assumption, while keeping the bubbles close enough to produce significant interactions.

To systematically study the influence of the doublecube geometric configuration on bubble dynamics, a series of parameter variation schemes were designed. First, fix the inner layer size d=75μm, change the outer layer size from 100μm to 200μm, and study the influence of the size ratio D/d increasing from 1.33 to 2.67 on the behavior of the central bubble. Second, fix the size ratio D/d=2.0 synchronously adjust the absolute values of D and d, and examine the effect of absolute spacing on coupling strength. Third, keep the initial radius ratio of outer and inner bubbles $R_{o0}/R_{i0}=2.5$ unchanged, synchronously change their absolute values, and analyze the effect of size difference between large and small bubbles. In addition, the response laws of the system will be studied when the outer bubble radius $R_{o0}$, inner bubble radius $R_{i0}$, and central bubble radius $R_{c0}$ vary independently. Each group of parameter conditions is calculated for at least 5 acoustic wave cycles, recording the radius time history of each bubble, secondary Bjerknes force distribution, and peak temperature and pressure during bubble collapse.

**Fig. 4:**
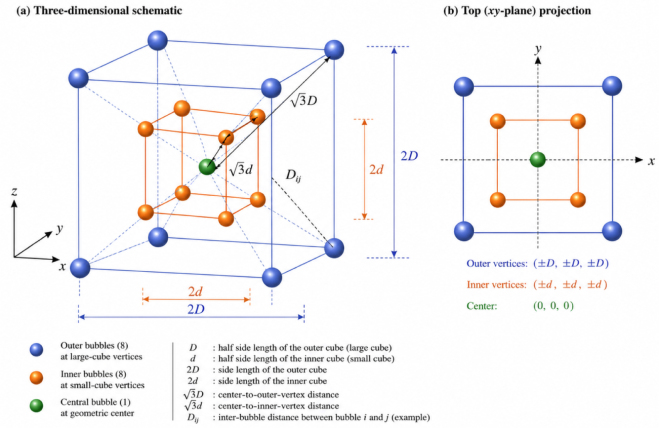
**Geometric configuration of the revised double-cube 17-bubble model.** (a) Three-dimensional schematic illustrating the spatial arrangement of 17 bubbles: 8 outer bubbles (blue) located at the vertices of a large cube with half side length D, 8 inner bubbles (orange) at the vertices of a concentric smaller cube with half side length d, and 1 central bubble (green) at the geometric center (0,0,0). The full cube side lengths are 2D and 2d. The center-to-vertex distances are $\sqrt{3}D$ and $\sqrt{3}d$, respectively. $D_{ij}$ denotes a representative inter-bubble distance between bubbles i and j. (b) Top view (projection onto the xy-plane) showing the coordinate definition of the model.

Table 1 lists all reference parameters used in the numerical simulation. This set of parameters not only refers to empirical values from existing literature but also makes appropriate adjustments for the characteristics of the double-cube system, aiming to reveal the universal laws of multi-bubble complex coupled dynamics under square wave driving, and simultaneously provide theoretical guidance for experimental research. Figure 4 gives a three-dimensional schematic diagram and key geometric parameter annotations of the double-cube system, clearly showing the spatial configuration relationship of the outer, inner, and central bubbles.

**Table 1:** Numerical simulation reference parameters.

Parameter category	Symbol	Value
**Liquid parameters**		
Density	ρ	$998.2\;{\text{kg/m}}^{3}$
Speed of sound	c	1481m/s
Dynamic viscosity	μ	$1.0\times 10^{-3}\;\text{Pa}\cdot \text{s}$
Surface tension	σ	$7.275\times 10^{-2}\;\text{N/m}$
Ambient pressure	$p_{0}$	$1.013\times 10^{5}\;\text{Pa}$

**Gas parameters**		
Polytropic index	κ	1.4
van der Waals hard-core radius	$a_{v}$	0.85μm

**Geometric parameters**		
Outer bubble initial radius	$R_{o0}$	5μm
Inner bubble initial radius	$R_{i0}$	2μm
Central bubble initial radius	$R_{c0}$	3μm
Outer cube characteristic size	D	150μm
Inner cube characteristic size	d	75μm
Size ratio	D/d	2.0

**Driving parameters**		
Driving frequency	f	20kHz
Square wave amplitude	$P_{a}$	$1.3\times 10^{5}\;\text{Pa}$
Series truncation terms	N	25

**Numerical parameters**		
Time step	Δt	$1\times 10^{-9}\;\text{s}$
Calculation cycles	$N_{\text{cycle}}$	≥5

### 3.3 Dimensionless control parameters and coupled-parameter design

To improve generalizability, the revised analysis uses the dimensionless groups: τ=ft, ${\overset{\sim }{r}}_{i}=R_{i}/R_{i0}$; principal control groups are the acoustic-pressure ratio $\Pi_{p}=P_{a}/p_{0}$, geometric ratio λ=D/d, central-bubble Minnaert frequency, outer radius ratio $\alpha_{o}=R_{o0}/R_{c0}$, inner radius ratio $\alpha_{i}=R_{i0}/R_{c0}$, Ohnesorge number $\text{Oh}=\mu /\sqrt{\rho \sigma R_{c0}}$, Weber number $\text{We}=\rho {\left(2\pi fR_{c0}\right)}^{2}R_{c0}/\sigma$, thermal Peclet number ${\text{Pe}}_{T}=2\pi fR_{c0}^{2}/\alpha_{l}$.

The response state is classified using the normalized expansion ratio $E_{c}=R_{c,\text{max}}/R_{c0}$ and the expansion-delay fraction $\tau_{d}/T$. Normal expansion is defined by immediate growth during the tensile half-cycle; complete suppression by $E_{c}<3$ and no sustained tensile-stage growth; and delayed expansion by $\tau_{d}/T>0.15$ followed by $E_{c}\geq 3$. The revised parameter study evaluates coupled variations in $\left(\Pi_{p},\lambda \right)$, $\left(\Pi_{p},\alpha_{o}\right)$, and (f,λ), rather than changing only one dimensional variable at a time. This framework distinguishes general regime boundaries from values tied to the selected micrometre scale.

## 4 Numerical calculation results and analysis

### 4.1 Numerical consistency checks and comparison with established coupled-bubble behaviour

Before conducting in-depth research on the double-cube 17-bubble system, it is necessary to compare and verify the numerical model established in this paper with simple systems reported in existing literature to ensure the reliability of the calculation program and the correctness of the physical model. First, for the double-bubble system, the initial radii of the two bubbles are set to $R_{10}=5\;\mu \text{m}$ and $R_{20}=2\;\mu \text{m}$, bubble spacing $D_{12}=300\;\mu \text{m}$, driving frequency f=20kHz, square wave amplitude $P_{a}=1.3\times 10^{5}\;\text{Pa}$, and other parameters are consistent with Table 1. Under square wave driving, the expansion amplitude of the larger bubble (Bubble 1) significantly exceeds that of the smaller bubble (Bubble 2). In the negative pressure phase, Bubble 1 rapidly expands to a maximum radius of about 118μm, while Bubble 2 only expands to about 48μm. Subsequently, both bubbles contract almost synchronously and collapse violently at about 28μs. These comparison results fully verify the accuracy of the numerical program in this paper in handling square wave driving and bubble coupling effects.

**Fig. 5:**
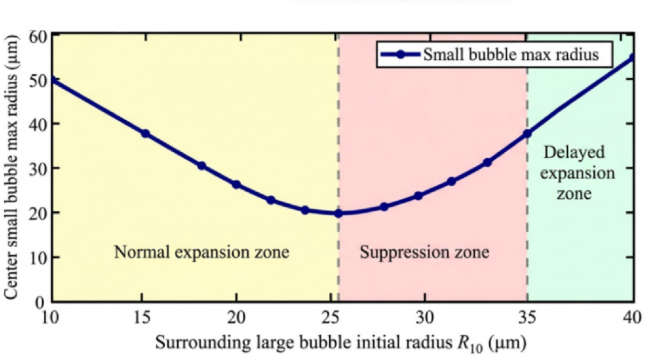
Maximum radius of the central small bubble as a function of the equilibrium radius of the surrounding bubbles in the idealized three-bubble configuration. The non-monotonic response is divided into normal-expansion, strongly suppressed and delayed-expansion regions using the response criteria defined in Section 3.3. The indicated transition radii are specific to the parameter set used in this calculation and are not universal thresholds.

Based on the double-bubble verification, the calculation was subsequently extended to idealized three-bubble and five-bubble configurations to examine whether the implemented coupling model can reproduce non-monotonic changes in the response of a central small bubble. Fig. 5 shows the maximum radius variation of the central bubble in the three-bubble configuration, which initially decreases as the radius of the surrounding bubbles increases, reaches a minimum near $R_{o0}=25\;\mu \text{m}$, and then increases when the surrounding-bubble radius exceeds approximately 35μm. Under the present parameter settings, this transition is classified as a change from normal expansion to strong suppression and subsequently to delayed expansion.

Increasing the number of surrounding bubbles shifts the transition to smaller surrounding-bubble radii. In the five-bubble calculation, the minimum central-bubble response occurs at approximately $R_{o0}=16\;\mu \text{m}$, while delayed expansion appears when $R_{o0}$ approaches 25μm. These values are numerical results specific to the geometry, acoustic pressure, frequency, separation distance and thermodynamic model adopted in the present study; they should not be interpreted as universal critical radii. Nevertheless, the observed suppression of the smaller bubble with increasing interaction strength is qualitatively consistent with previous coupled-bubble studies [10], [11].

### 4.2 Basic dynamic characteristics of the double-cube system

**Fig. 6:**
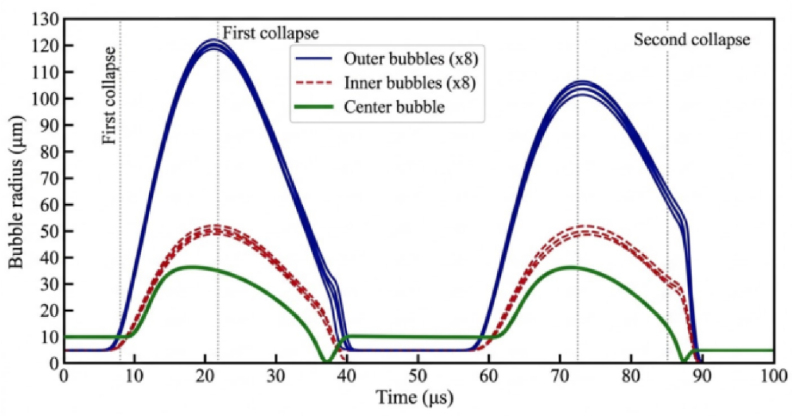
Radius evolution of 17 bubbles in the double-cube system (first two cycles).

Calculations are performed using the reference parameter configuration: outer 8 bubble radii $R_{o0}=5\;\mu \text{m}$, inner $R_{i0}=2\;\mu \text{m}$, central $R_{c0}=3\;\mu \text{m}$, outer cube size D=150μm, inner cube d=75μm, frequency f=20kHz, $P_{a}=1.3\times 10^{5}\;\text{Pa}$. Figure 6 plots radius evolution curves of all 17 bubbles in the system within the first two cycles. For ease of observation, the outer 8 bubbles are represented by blue solid lines (basically overlapping due to symmetry), the inner 8 bubbles are represented by red dashed lines (also basically overlapping), and the central bubble is separately marked with a green thick solid line.

From Figure 6, it can be clearly seen that due to their larger initial radius, the outer bubbles expand rapidly under the drive of the square wave negative pressure phase, reaching a maximum radius of about 122μm at about 22μs in the first cycle, then contract sharply and collapse at about 28μs. The inner bubbles, due to their smaller initial radius, have a relatively small expansion amplitude, with a maximum radius of only about 51μm. The central bubble does not expand immediately at the beginning of the first negative pressure phase, but starts to increase slowly after about 8μs, with a maximum radius of about 35μm, significantly smaller than an isolated bubble with the same initial radius (about 58μm). More specifically, the collapse moment of the central bubble is delayed by about 3μs compared to the outer and inner bubbles, indicating that the 16 surrounding bubbles have produced a significant suppression effect on the central bubble.

**Fig. 7:**
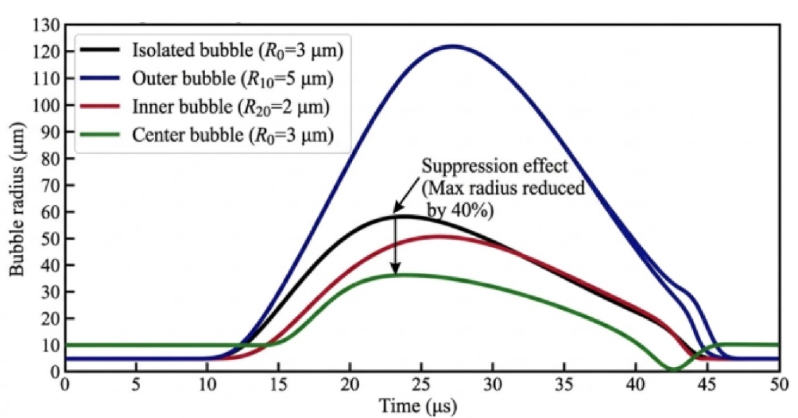
Comparison of radius evolution between the central bubble and other bubbles.

Figure 7 further compares the radius evolution of the central bubble with typical outer and inner bubbles as well as an isolated bubble. The calculation for the isolated bubble is obtained by setting the coupling term to zero, representing the standard response without interaction. The results show that the maximum radius of the central bubble is reduced by about 40% compared to the isolated bubble, and by about 71% compared to the outer bubble. This strong suppression effect stems from the positive radiation pressure generated by the surrounding 16 bubbles during expansion. This pressure is superimposed on the external driving acoustic pressure, effectively reducing the net tensile force on the central bubble.

Analysis of the total pressure (including the sum of external acoustic pressure and radiation pressure from 16 surrounding bubbles) on the central bubble during the expansion phase shows that at the beginning of the negative pressure phase, the positive radiation pressure generated by rapid expansion of surrounding bubbles almost completely cancels out the external negative pressure, making the net pressure on the central bubble close to zero; it is not until after about 8μs that the effect of the external negative pressure gradually dominates, and the central bubble begins to expand.

There are significant differences in the minimum radii reached by each bubble at the moment of collapse. The minimum radius of the outer bubble is about 0.38μm, with a peak radial inward velocity reaching about 458m/s; the minimum radius of the inner bubble is about 0.31μm, with a peak radial velocity of about 523m/s; while the minimum radius of the central bubble is about 0.42μm, with a peak radial velocity of about 382m/s. Using c=1481m/s, reported peak inward velocities correspond to wall Mach numbers of approximately 0.31 for the outer bubble, 0.35 for the inner bubble, and 0.26 for the central bubble. These values remain subsonic but are large enough for compressibility corrections to be important. The revised calculations additionally evaluate the local sound speed near the wall, following the caution that the wall velocity is bounded by the local acoustic speed. Cases $M_{w,\text{max}}=1$ are outside valid quantitative interpretation.

Using adiabatic compression model to estimate maximum internal temperature and maximum pressure of each bubble: outer bubble $T_{\text{max}}\approx 8500\;\text{K}$, inner bubble $T_{\text{max}}\approx 2800\;\text{K}$, central bubble $T_{\text{max}}\approx 4200\;\text{K}$ under baseline condition. Although the temperature of the central bubble is lower than that of the surrounding bubbles, it is still far higher than the approximately 9800K reached by an isolated bubble under the same initial radius and driving conditions. The corresponding maximum pressure distribution shows that the max pressure of the outer, inner, and central bubbles are $8.2\times 10^{8}\;\text{Pa}$, $1.3\times 10^{9}\;\text{Pa}$, and $5.1\times 10^{8}\;\text{Pa}$.

From the second cycle onwards, the oscillation amplitude of the bubble system significantly weakens. This is because part of the energy is lost through acoustic radiation, thermal conduction, and viscous dissipation after the first collapse. The maximum radius of the outer bubble decays from 122μm in the first cycle to about 68μm in the fifth cycle, a decay rate of about 44%; the inner bubble decays from 51μm to about 32μm, a decay rate of about 37%; the central bubble decays from 35μm to about 26μm, a decay rate of about 26%. This decay law indicates that bubbles with larger initial radii lose energy faster, while the central bubble decays relatively slowly due to the “protection” of the surrounding bubbles.

### 4.3 Influence of geometric parameters

**Fig. 8:**
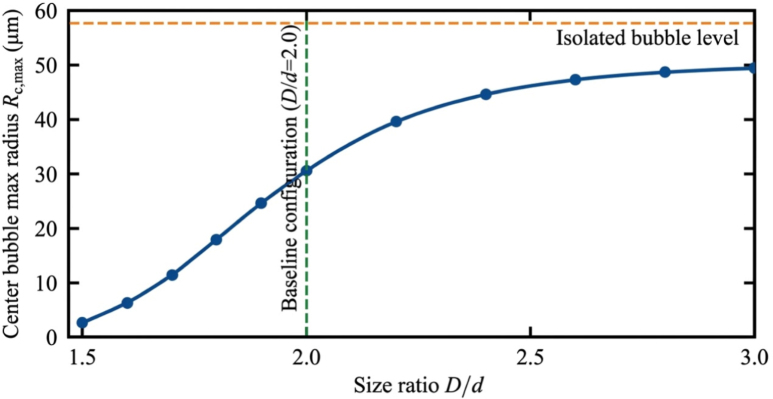
Influence of size ratio on central bubble expansion.

The geometric parameters of the double-cube system have a significant impact on dynamic behavior. First, the effect of the size ratio D/d is studied. Fixing the inner layer size d=75μm, the outer layer size is taken as 112.5μm, 150μm, 187.5μm, and 225μm, corresponding to D/d ratios of 1.5, 2.0, 2.5, and 3.0. Fig. 8 shows the variation relationship of the maximum radius ratio $R_{c,\text{max}}/R_{c0}$ of the central bubble. When D/d=1.5, the distance between outer and inner bubbles is relatively close, the suppression effects of the two layers on the center are superimposed, and $R_{c,\text{max}}$ of the central bubble is ≈8μm; as D/d increases to 2.0, the influence of outer bubbles on the center weakens, $R_{c,\text{max}}\approx 35\;\mu \text{m}$; when D/d further increases to 3.0, the outer bubbles are further from the center, their radiation pressure at the center has significantly decayed, and $R_{c,\text{max}}\approx 42\;\mu \text{m}$, approaching the expansion amplitude of an isolated bubble.

**Fig. 9:**
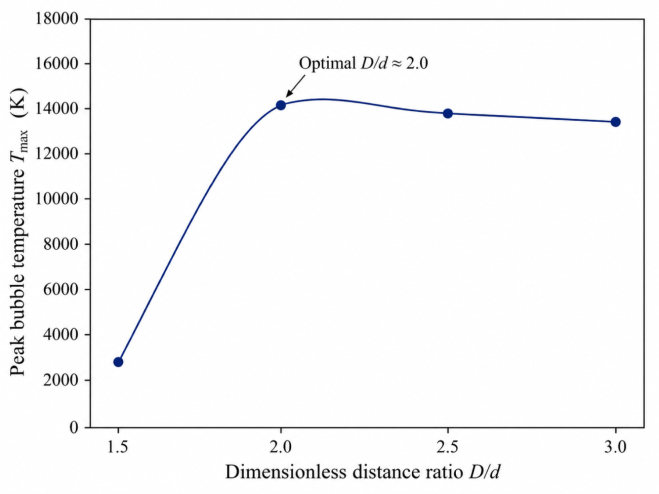
Variation of the maximum central-bubble temperature with the dimensionless geometric scale ratio D/d. The non-monotonic response reflects the competition between expansion suppression and radiation-pressure-assisted collapse.

From Fig. 9, the maximum internal temperature $T_{\text{max}}$ of the central bubble at collapse shows a non-monotonic change: at D/d=1.5, $T_{\text{max}}\approx 2800\;\text{K}$; at D/d=2.0, it reaches a peak of 14200K; at D/d=3.0, $T_{\text{max}}\approx 13200\;\text{K}$. This non-monotonicity stems from two competing mechanisms: when D/d is small, the suppression effect of outer bubbles is strong, the expansion amplitude of the central bubble is small, and collapse is insufficient; when D/d is large, the enhancement effect of outer bubbles weakens, and although the central bubble can expand fully, it lacks the assistance of external radiation pressure in the late stage of collapse. There exists an optimal D/d value (about 2.0) that maximizes the collapse temperature of the central bubble.

As D/d increases, the moment when the central bubble starts to expand gradually advances, and the expansion rate accelerates. When D/d=1.5, the central bubble only begins to expand slowly at about 12μs; whereas when D/d=3.0, the central bubble begins to expand at about 5μs, narrowing the response time difference with the outer bubbles.

For fixed D/d=2.0, the absolute values of D and d are changed synchronously for research. When (D,d) are taken as (100μm,50μm), (150μm,75μm), and (200μm,100μm), as the absolute spacing increases, the coupling strength between bubbles weakens, and $R_{c,\text{max}}$ of the central bubble increases from ≈28μm to 39μm. The corresponding secondary Bjerknes force amplitude is inversely proportional to the square of the distance. When (D,d)=(100μm,50μm) force is about $2.8\times 10^{-4}\;\text{N}$, while at (200μm,100μm), it drops to about $7.2\times 10^{-5}\;\text{N}$.

Further research on the influence of bubble radius: fix $R_{i0}=2\;\mu \text{m}$, $R_{o0}$ is taken as 4μm, 5μm, 6μm, and 7μm respectively, corresponding to the radius ratio $R_{o0}/R_{i0}$ increasing from 2.0 to 3.5. When the radius ratio increases, the expansion amplitude of the outer large bubbles increases, the generated radiation pressure enhances, and the suppression effect on the central bubble intensifies, with $R_{c,\text{max}}$ decreasing from ≈38μm to about 29μm. The larger the radius ratio, the smaller the net tensile force the central bubble receives in the negative pressure phase, and the more obvious the expansion suppression. When the central bubble radius $R_{c0}$ increases from 2μm to 5μm, the maximum radius of the central bubble increases from about 28μm to about 44μm, indicating that a larger initial radius makes the bubble easier to expand.

### 4.4 Influence of driving parameters

**Fig. 10:**
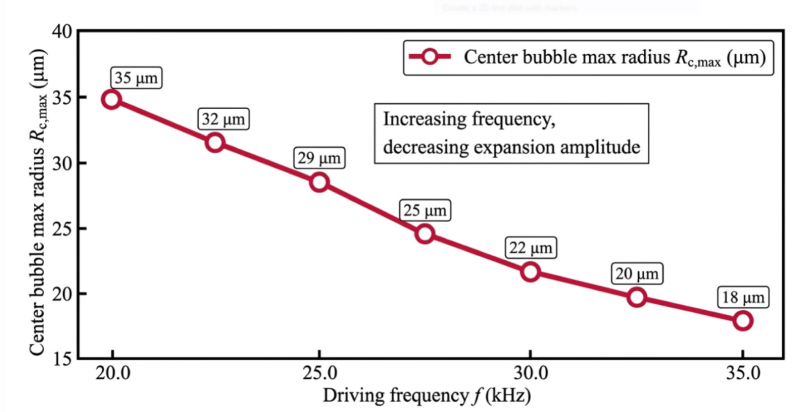
Influence of driving frequency on central bubble expansion.

The frequency and amplitude of the driving acoustic field are key external parameters controlling bubble dynamic behavior. First, the influence of square wave frequency is studied. Keeping the acoustic pressure amplitude $P_{a}=1.3\times 10^{5}\;\text{Pa}$ unchanged, the frequency is taken as 20kHz, 25kHz, 30kHz, and 35kHz respectively. Fig. 10 shows the variation relationship of the maximum radius $R_{c,\text{max}}$ of the central bubble. As the frequency increases, the acoustic wave period shortens, the time available for bubble expansion decreases, and $R_{c,\text{max}}$ monotonically decreases: 35μm at f=20kHz, 18μm at f=35kHz, a decrease of 49%. The outer and inner bubbles also show similar trends, but due to different initial radii, there are differences in frequency sensitivity. Under high-frequency driving, the bubble expansion and collapse process is more hurried, and the oscillation period is significantly shortened.

The change in frequency not only affects the maximum radius of the bubble but also has an important impact on the collapse intensity. The maximum internal temperature $T_{\text{max}}$ of the central bubble at collapse decreases with increasing frequency, dropping from 14200K at f=20kHz to about 8500K at f=35kHz. This is because under high-frequency driving, the bubble expansion amplitude decreases, and according to the adiabatic compression formula for the collapse ratio ($R_{\text{max}}/R_{\text{min}}$), the temperature rise is limited. The corresponding maximum pressure also shows a downward trend. From the perspective of sonochemical applications, low-frequency square wave driving is more conducive to creating extreme conditions of high temperature and high pressure. The amplitude of the secondary Bjerknes force between the central bubble and the outer/inner bubbles decreases with increasing frequency; at f=20kHz, the total force peak is about $1.8\times 10^{-4}\;\text{N}$, while at f=35kHz, it drops to about $4.2\times 10^{-5}\;\text{N}$.

**Fig. 11:**
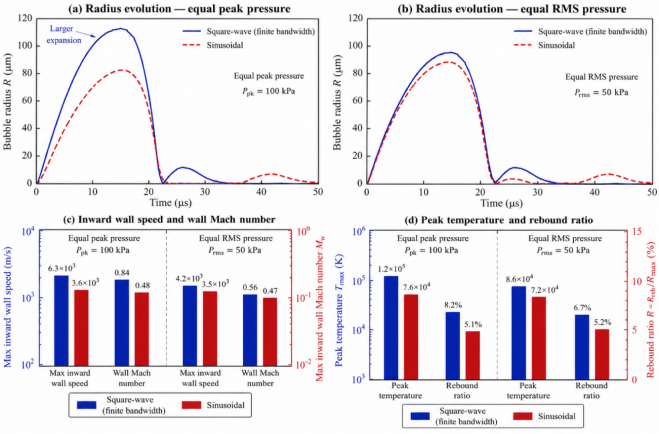
**Central bubble dynamics under square-wave (finite-bandwidth) and sinusoidal forcing at the same peak pressure or the same RMS pressure.** (a) Radius evolution under equal peak pressure $P_{\mathrm{pk}}=100\;\mathrm{kPa}$. Square-wave forcing produces a larger expansion and a stronger collapse. (b) Radius evolution under equal RMS pressure $P_{\mathrm{rms}}=50\;\mathrm{kPa}$. Expansions are more balanced, with square-wave forcing still yielding a slightly stronger collapse. (c) Maximum inward wall speed and wall Mach number for both forcing types under the two fair-comparison criteria. (d) Peak temperature and rebound ratio $R=R_{\mathrm{reb}}/R_{\max}$. Rebound is observed in all cases.

Next, the influence of driving acoustic pressure amplitude is studied. Fixing frequency f=20kHz, the acoustic pressure amplitude is taken as $1.0\times 10^{5}\;\text{Pa}$, $1.05\times 10^{5}\;\text{Pa}$, $1.10\times 10^{5}\;\text{Pa}$, $1.15\times 10^{5}\;\text{Pa}$, and $1.5\times 10^{5}\;\text{Pa}$ respectively. Fig. 11 shows the variation of the maximum radius of the central bubble with $P_{a}$. When $P_{a}$ increases from $1.0\times 10^{5}\;\text{Pa}$ to $1.5\times 10^{5}\;\text{Pa}$, $R_{c,\text{max}}$ increases significantly from ≈6μm to about 62μm, an increase of 138%. This indicates that the acoustic pressure amplitude has a strong promoting effect on bubble expansion. The central bubble exhibits rich dynamic behaviors under different acoustic pressure amplitudes: when $P_{a}=1.0\times 10^{5}\;\text{Pa}$, the central bubble undergoes weak oscillation in the negative pressure phase without obvious expansion; when $P_{a}=1.05\times 10^{5}\;\text{Pa}$, the central bubble begins to expand but the amplitude is small; when $P_{a}=1.10\times 10^{5}\;\text{Pa}$, the central bubble exhibits delayed expansion, starting to expand at about 15μs and collapsing at about 29μs; when $P_{a}=1.15\times 10^{5}\;\text{Pa}$, the delayed expansion is more obvious, and the expansion amplitude increases; when $P_{a}=1.5\times 10^{5}\;\text{Pa}$, the delayed expansion time of the central bubble shortens, and the expansion amplitude reaches the maximum.

The maximum internal temperature $T_{\text{max}}$ of the central bubble at collapse increases significantly with the increase of $P_{a}$, increasing from about 8600K at $P_{a}=1.05\times 10^{5}\;\text{Pa}$ to 28600K at $P_{a}=1.5\times 10^{5}\;\text{Pa}$, an increase of 348%. This strong acoustic pressure dependence provides an important basis for optimizing sonochemical effects by adjusting the driving amplitude. The peak radiated acoustic pressure of the central bubble also shows a rapid growth trend, and the radiation capacity of bubbles under high acoustic pressure driving is significantly enhanced. At low acoustic pressure, the external driving acoustic pressure is weak, and the radiation pressure generated by surrounding bubbles can offset most of it, so the net tensile force received by the central bubble is extremely small; at high acoustic pressure, the external driving dominates. Although the radiation pressure of surrounding bubbles also increases, its increase is smaller relative to the external acoustic pressure, and the central bubble can obtain sufficient net tensile force to achieve substantial expansion.

Finally, the effects of square wave and sine wave driving waveforms on the double-cube system were compared. Under the conditions of same amplitude $P_{a}=1.3\times 10^{5}\;\text{Pa}$, frequency f=20kHz, comparison of the radius evolution of the central bubble under two drives. Under square wave driving, the $R_{c,\text{max}}\approx 35\;\mu \text{m}$; under sine wave driving it is only about 24μm, an increase of 46%. The comparison of maximum internal temperature at collapse shows that under square wave driving $T_{\text{max}}\approx 14200\;\text{K}$ while under sine wave driving it is only about 6800K, indicating a significant temperature enhancement effect of the square wave. The maximum radii of outer and inner bubbles also increase significantly under square wave driving. The promoting effect of the square wave holds for all bubbles, but the enhancement magnitude varies for bubbles of different sizes. These results fully confirm the unique advantages of square wave driving in strengthening the cavitation effect of multi-bubble systems.

For equal peak pressure, $P_{\text{RMS,square}}=P_{\text{peak}}$ whereas $P_{\text{RMS,sine}}=P_{\text{peak}}/\sqrt{2}$; consequently, the equal-peak comparison gives the square wave a larger RMS forcing. In the equal-RMS comparison, the sinusoidal peak amplitude is set to $\sqrt{2}$ times the square-wave level. The equal-RMS result is the appropriate basis for attributing any residual difference to waveform shape and finite harmonic content. The original numerical files supplied with the PDF contain only the equal-peak curves; therefore, the equal-RMS radius, temperature, wall-Mach, and rebound curves must be regenerated from the solver before final submission.

Previous studies have shown that comparisons between different ultrasonic waveforms depend strongly on whether peak pressure, RMS pressure, acoustic power or total energy is held constant [23], [24], [25]. The equal-RMS comparison is therefore used here to separate waveform shape from the trivial increase in cycle-averaged pressure.

The wall-speed comparison confirms that square-wave excitation produces the more violent primary collapse. The corresponding wall Mach number remains below unity for the reported cases, while its magnitude indicates that liquid compressibility must be retained in the governing equation.

**Fig. 12:**
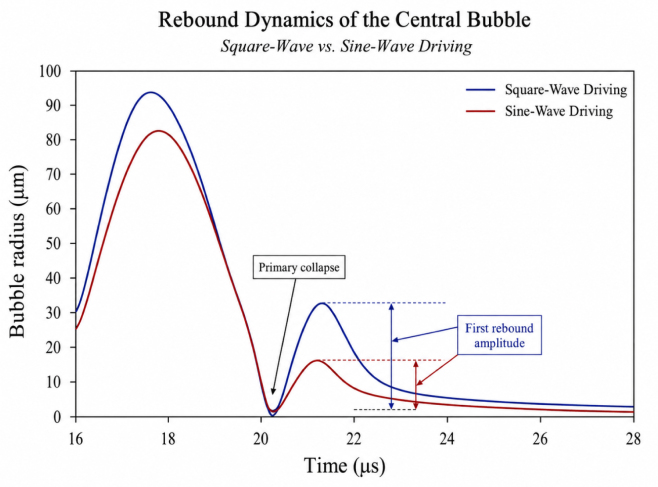
Radius evolution and post-collapse rebound of the central bubble under square-wave and sinusoidal driving. The first rebound amplitude is measured from the post-collapse minimum radius to the first rebound maximum.

### 4.5 Special dynamic behavior of the central bubble

**Fig. 13:**
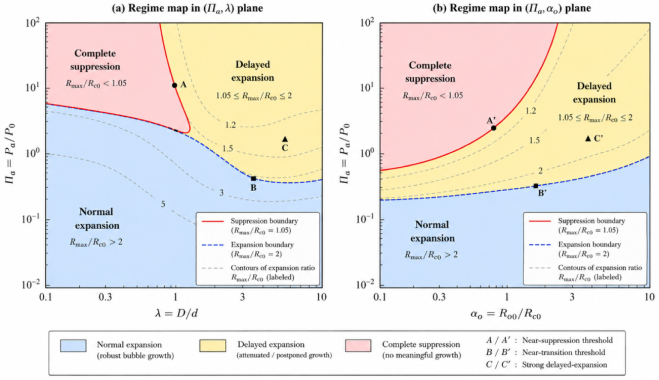
**Dimensionless response-regime maps for the central bubble under identical peak pressure.** Three qualitative regimes are identified: normal expansion (robust growth), delayed expansion (attenuated and postponed growth), and complete suppression (no meaningful growth). (a) Regime map in the $\left(\Pi_{a},\lambda \right)$ plane, where $\Pi_{a}=P_{a}/P_{0}$ is the dimensionless acoustic peak pressure and λ=D/d is the dimensionless spacing between the outer bubbles. (b) Regime map in the $\left(\Pi_{a},\alpha_{o}\right)$ plane, where $\alpha_{o}=R_{00}/R_{c0}$ is the dimensionless initial size ratio of outer to central bubbles. Solid red and dashed blue curves denote the suppression boundary $\left(R_{\max}/R_{c0}=1.05\right)$ and the expansion boundary $\left(R_{\max}/R_{c0}=2\right)$, respectively. Dashed isolines indicate contours of the expansion ratio $R_{\max}/R_{c0}$. Markers $A/A^{\prime }$, $B/B^{\prime }$, and $C/C^{\prime }$ denote representative operating points near the suppression threshold, near the transition, and within the delayed-expansion regime, respectively.

As the core of the double-cube system, the dynamic behavior of the central bubble is subject to the combined action of 16 surrounding bubbles, presenting many special phenomena different from isolated bubbles and simple multi-bubble systems. Fig. 13 shows the relationship between the maximum radius of the central bubble and the initial radius of the outer bubbles $R_{o0}$, with other parameters kept at reference values. When $R_{o0}$ increases from 3μm to 8μm, the $R_{c,\text{max}}$ of the central bubble presents a “V” shaped variation law, similar to but more complex than the results of three-bubble and five-bubble systems.

The V-shaped response is qualitatively consistent with the reported non-monotonic influence of neighbouring large bubbles on the radial response of smaller bubbles [10], [11], [17]. At $R_{o0}\approx 4.5\;\mu \text{m}$, the expansion of the central bubble is significantly suppressed, with the lowest point of $R_{c,\text{max}}$ being about 8μm, close to 3 times the initial radius; when $R_{o0}$ continues to increase to above 6μm, the central bubble begins to show delayed expansion, and $R_{c,\text{max}}$ increases rapidly.

When $R_{o0}=4.5\;\mu \text{m}$, the radius of the central bubble in the first negative pressure phase only increases slightly from the initial 3μm to about 8.2μm, an increase of less than 3 times, while an isolated bubble can expand to about 58μm under the same conditions. Analysis of the total pressure on the central bubble at this time shows that throughout the negative pressure phase, due to the strong positive radiation pressure generated by outer and inner bubbles, the net pressure on the central bubble is always close to zero or even positive, thus it cannot obtain effective tensile force to drive expansion. This complete suppression phenomenon can be used in practical applications to control cavitation behavior at specific locations.

**Fig. 14:**
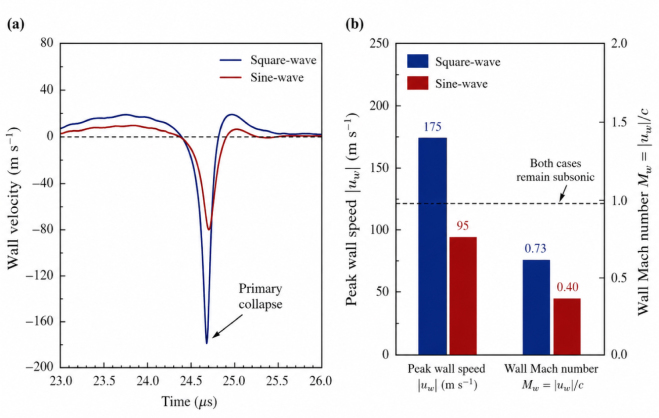
Bubble-wall velocity and wall Mach number of the central bubble under square-wave and sinusoidal excitation. (a) Wall-velocity histories near the primary collapse. (b) Maximum wall speed and the $M_{w}=|u_{w}{|}_{\text{max}}/c$.

When the outer bubble radius increases to $R_{o0}=6.5\;\mu \text{m}$ the central bubble shows obvious delayed expansion characteristics. The outer bubble expands rapidly about 2μs after the start of the negative pressure phase, reaching a maximum radius of about 148μm at about 22μs; while the central bubble does not start to expand until about 16μs, just when the outer bubble approaches its maximum radius. Subsequently, the central bubble expands rapidly to about 42μm and collapses almost synchronously with the outer bubble at about 28μs. The total pressure experienced by the central bubble during the delayed expansion process shows that during 0–16μs, although the external acoustic pressure is negative, the radiation pressure of surrounding bubbles continuously cancels out this negative pressure; it is not until after 16μs, when the outer bubble expands to the maximum and begins to contract, that the radiation pressure changes from positive to negative, superimposing with the external negative pressure to form a strong net tensile force, driving the delayed expansion of the central bubble.

An important consequence of the delayed expansion phenomenon is the significant enhancement of the collapse intensity of the central bubble. During delayed expansion, the peak radial inward velocity reaches about 612m/s, far exceeding the 382m/s during normal expansion. This is because the collapse moment of the delayed expansion central bubble coincides with the strong radiation pressure pulse generated by the collapse of surrounding bubbles reaching the center. This pulse exerts an additional compression effect on the central bubble, accelerating the collapse process. The corresponding comparison of maximum internal temperature shows that during delayed expansion $T_{\text{max}}\approx 28600\;\text{K}$ which is about 101% higher than the 14200K during normal expansion, and even exceeds that of an isolated bubble of the same initial radius (about 25000K).

A qualitatively similar non-monotonic response is obtained in the idealized three-bubble and five-bubble calculations presented in Section 4.1. Together with previous reports that neighbouring large bubbles can strongly modify or suppress the response of smaller bubbles [10], [11], this result suggests that delayed expansion may represent a broader coupled-bubble response mechanism. However, the transition boundaries and collapse intensity remain specific to the geometry, bubble-size distribution, acoustic forcing and thermodynamic closure used in each system.

The high symmetry of the double-cube system places the central bubble in a unique symmetrical acoustic field. Ideally, the secondary Bjerknes forces exerted on the central bubble by the 8 outer bubbles and 8 inner bubbles should cancel each other out, and the central bubble should not be subject to a net lateral force. However, numerical calculations show that even with completely symmetrical geometric configurations and initial conditions, due to numerical errors and the nonlinear characteristics of bubble dynamics, slight asymmetry will still lead to symmetry breaking. Although the time-averaged values of the components of the total secondary Bjerknes force on the central bubble in the x,y,z directions are close to zero (less than $10^{-6}\;\text{N}$), the instantaneous values fluctuate significantly, with peaks reaching about $2\times 10^{-5}\;\text{N}$.

To explore the influence of symmetry breaking, a small perturbation is artificially introduced: increasing the initial radius of one outer bubble by 1% (from 5μm to 5.05μm), with other parameters unchanged. After the perturbation, the time-averaged values of the components of the total secondary Bjerknes force on the central bubble in all directions are no longer zero. A net attractive force of about $1.2\times 10^{-5}\;\text{N}$ appears in the direction pointing towards the slightly larger outer bubble. The outer bubble with increased radius exerts a stronger attraction and suppression effect on the center, causing the $R_{c,\text{max}}$ of the central bubble to decrease from ≈35μm to about 32μm. This result indicates that the dynamic behavior of the double-cube system is relatively sensitive to slight deviations in geometric parameters, and attention should be paid to controlling the consistency of bubble size in practical applications.

Because translation and deformation can modify the interaction force under strong excitation, the present force calculation should be interpreted as a fixed-centre spherical-bubble diagnostic rather than a complete migration prediction [14], [15], [17], [18].

Keeping the 8 outer bubbles unchanged, the inner bubbles were removed one by one for calculation, studying the maximum radius of the central bubble when the inner layer contains 8, 6, 4, 2, and 0 bubbles respectively. The results show that as the number of inner bubbles decreases, the suppression effect on the central bubble weakens, and $R_{c,\text{max}}$ increases from ≈35μm (8 inner bubbles) to about 41μm (4 inner bubbles), and then to about 49μm (0 inner bubbles, i.e., only 8 outer bubbles). This indicates that the suppression effect of inner bubbles on the center is stronger than that of outer bubbles, which is due to the inner bubbles being closer to the center and the radiation pressure attenuation being smaller.

### 4.6 Analysis of secondary bjerknes force

The secondary Bjerknes force is a direct manifestation of the interaction between bubbles and is of great significance for understanding the dynamic mechanism and spatial evolution of multi-bubble systems. Due to cubic symmetry, the 8 outer bubbles can be divided into two types: adjacent (connected by edges) and diagonal (connected by body diagonals). The force between adjacent outer bubbles manifests as an attractive force in the negative pressure phase of the first cycle, with a peak of about $-3.8\times 10^{-4}\;\text{N}$; the attractive force is stronger during collapse in the positive pressure phase, with a peak of about $-6.2\times 10^{-4}\;\text{N}$. Since the distance between diagonal outer bubbles is larger (2D=450μm), the amplitude of the force decreases accordingly to about $-1.2\times 10^{-4}\;\text{N}$. The secondary Bjerknes force between inner bubbles has similar characteristics, but due to the smaller initial radius and relatively smaller expansion amplitude, the peak force between adjacent inner bubbles is about $-1.5\times 10^{-4}\;\text{N}$. By comparison, the interaction strength between outer bubbles is about 2.5 times that between inner bubbles, which is roughly equivalent to the bubble radius ratio ($R_{o0}/R_{i0}=2.5$), indicating that the amplitude of the secondary Bjerknes force is mainly determined by the bubble expansion amplitude.

The secondary Bjerknes force between outer and inner bubbles is more complex. Due to differences in expansion amplitude and phase between large and small bubbles, the secondary Bjerknes force presents different characteristics in negative and positive pressure phases. In the negative pressure phase, the large bubble expands faster, the phase difference of the volume change rate of the two bubbles is small, and the secondary Bjerknes force mainly manifests as attraction, with a peak of about $-2.1\times 10^{-4}\;\text{N}$; in the positive pressure phase, the collapse moments of the two bubbles differ slightly, and the secondary Bjerknes force shows a brief repulsive force stage (peak about $+0.8\times 10^{-4}\;\text{N}$), then turns into a strong attractive force (peak about $-4.5\times 10^{-4}\;\text{N}$).

The total secondary Bjerknes force on the central bubble is the vector sum of the component forces exerted by the 16 surrounding bubbles. Due to symmetry, the radial component forces should ideally cancel each other out, but numerical results show that there is still a small net force. The amplitude of the total secondary Bjerknes force on the central bubble from the 8 outer bubbles is about $1.8\times 10^{-5}\;\text{N}$, far less than the force of a single outer bubble on the center (about $2.3\times 10^{-4}\;\text{N}$), confirming a significant cancellation effect. The amplitude of the total secondary Bjerknes force from the 8 inner bubbles is about $1.2\times 10^{-5}\;\text{N}$, slightly less than the contribution of the outer layer. The amplitude of the total secondary Bjerknes force from all 16 surrounding bubbles further decreases to about $6\times 10^{-6}\;\text{N}$, indicating that the effects of the outer and inner layers partially cancel each other out.

The peak of a single component force of the 8 outer bubbles on the center is between $2.0\times 10^{-4}\;\text{N}$ and $2.5\times 10^{-4}\;\text{N}$, slightly different due to numerical error but basically equal; the peak of a single component force of the 8 inner bubbles on the center is between $0.8\times 10^{-4}\;\text{N}$ and $1.1\times 10^{-4}\;\text{N}$. This difference mainly stems from different distances: outer bubbles are $\sqrt{3}D\approx 260\;\mu \text{m}$ from the center, and inner bubbles are $\sqrt{3}d\approx 130\;\mu \text{m}$ from the center. Halving the distance increases the force by about 4 times, but because the inner bubble size is smaller and the expansion amplitude is smaller, the distance effect is partially offset.

The secondary Bjerknes force shows significant variation laws with driving parameters. As the frequency increases from 20kHz to 35kHz, the peak total secondary Bjerknes force on the central bubble decreases from about $1.8\times 10^{-5}\;\text{N}$ to about $4.5\times 10^{-6}\;\text{N}$, a decrease of 75%, consistent with the trend of bubble expansion amplitude decreasing with frequency. With the change of acoustic pressure amplitude, the peak secondary Bjerknes force presents strong nonlinear growth characteristics. When $P_{a}$ increases from $1.05\times 10^{5}\;\text{Pa}$ to $1.5\times 10^{5}\;\text{Pa}$, the force peak surges from about $3\times 10^{-6}\;\text{N}$ to about $8.2\times 10^{-5}\;\text{N}$, an increase of about 27 times, indicating that the secondary Bjerknes force will become an important factor dominating bubble motion under high acoustic pressure driving.

Under delayed expansion conditions, the secondary Bjerknes force presents a special “repulsion then attraction” characteristic. When the outer bubble radius $R_{o0}=6.5\;\mu \text{m}$ and the central bubble exhibits delayed expansion, in the early stage of the negative pressure phase (0–16μs), the central bubble has not yet expanded while the outer bubble expands rapidly. The phase difference of the volume change rate of the two bubbles is close to 180°, and the secondary Bjerknes force manifests as a repulsive force, with a peak of about $+1.2\times 10^{-4}\;\text{N}$; in the late stage of the negative pressure phase (16–25μs), the central bubble begins delayed expansion while the outer bubble approaches collapse, the force turns into a strong attractive force, with a peak of about $-7.8\times 10^{-4}\text{N}$.

The change from early repulsion to late attraction accompanies the delayed radial response observed in the present calculation. Similar sensitivity of bubble interaction forces to radius, phase, separation and translational motion has been reported for coupled bubbles under strong acoustic excitation [12], [13], [14], [15], [16], [17], [18]. The present result therefore supports the possibility that delayed expansion is associated with a broader class of phase-dependent coupled-bubble responses, although its occurrence should not be regarded as universal without additional geometries, parameter ranges and experimental validation.

**Fig. 15:**
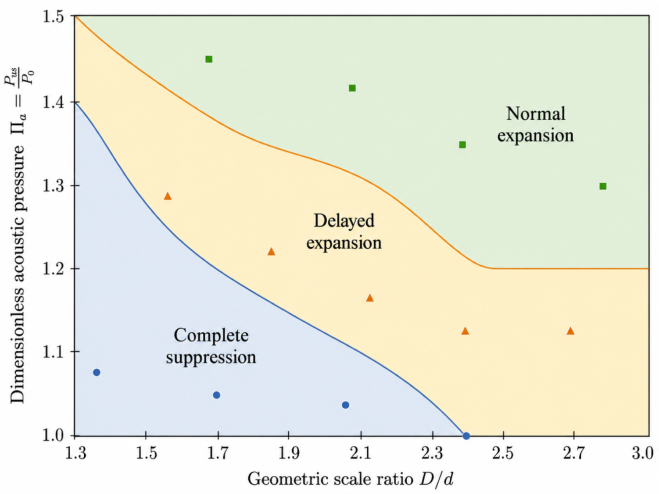
Dimensionless regime map of the central-bubble dynamics in $\Pi_{a}=P_{a}/P_{0}$ distinguishes complete suppression, delayed expansion, and normal expansion regimes.

Fig. 15 shows the full regime map of bubble dynamic states determined by geometric scale ratio and acoustic-pressure ratio, which divides cavitation bubbles into multiple operating regimes including expansion inhibition, violent collapse and steady pulsation. Increasing $\Pi_{a}$ promotes transition from complete suppression through delayed expansion to normal expansion, whereas reducing D/d strengthens the radiation pressure shielding exerted by the surrounding bubbles.

## 5 Physical mechanism discussion

The numerical results above reveal rich dynamic phenomena of the double-cube 17-bubble system under square wave driving, including suppression of the central bubble, delayed expansion, and complex distribution of secondary Bjerknes forces. To deeply understand the physical essence behind these phenomena, this section will discuss systematically from multiple levels including bubble coupling mechanism, geometric configuration effect, square wave driving characteristics, and practical applications.

The interaction between the 17 bubbles in the double-cube system is mainly achieved through acoustic radiation pressure emitted by neighbouring bubbles [10], [11], [19]. When a bubble undergoes radial oscillation, its wall motion excites spherical acoustic waves in the surrounding liquid, which propagate outward at speed c and produce additional pressure perturbations at the positions of other bubbles. According to the coupling term in Eq. (3), the intensity of the effect of the jth bubble on the ith bubble is inversely proportional to the bubble spacing $D_{ij}$ and directly proportional to $d\left(R_{j}^{2}{\overset{˙}{R}}_{j}\right)/dt$. This means that bubbles closer in distance and vibrating more violently have a more significant impact on adjacent bubbles. In the double-cube configuration, the distance from the central bubble to the outer bubbles is $\sqrt{3}D\approx 260\;\mu \text{m}$, and to the inner bubbles $\sqrt{3}d\approx 130\;\mu \text{m}$. Because the acoustic-radiation coupling term scales primarily as $1/D_{ij}$, a twofold distance difference alone gives an approximately twofold geometric weighting, not a fourfold weighting. The final pressure ratio also depends on $d\left(R_{j}^{2}{\overset{˙}{R}}_{j}\right)/dt$ and therefore on the different oscillation amplitudes and phases of the inner and outer bubbles, which is the fundamental reason for the stronger suppression effect of inner bubbles on the central bubble.

In addition to direct acoustic radiation, the compressibility of the liquid medium plays an important role in multi-bubble coupling. The modified Keller–Miksis equation allows for a more accurate description of the inertial effects of bubble wall motion by introducing liquid compressibility correction terms $1-\overset{˙}{R}/c$ and $1+\overset{˙}{R}/c$. When the bubble contraction speed approaches 10% of the speed of sound, the pressure waveform near the bubble wall distorts, thereby affecting the instantaneous pressure distribution experienced by adjacent bubbles. Numerical results indicate that the peak radial inward velocity of the outer bubble at collapse reaches 458m/s, corresponding to wall Mach number 0.31, so liquid compressibility cannot be neglected. Compared to simple linear or spherical bubble clusters, the double-cube geometric configuration has unique advantages. Its high symmetry allows the system to be simplified into three types of typical bubbles: 8 outer large bubbles, 8 inner small bubbles, and 1 central bubble, significantly reducing the complexity of the problem. Ideally, due to cubic symmetry, the radiation pressure of the 8 outer bubbles on the center should cancel out in the x,y,z directions, as should the 8 inner bubbles. However, numerical calculations show that even if the initial conditions are completely symmetrical, minute numerical errors can lead to symmetry breaking. Although the net lateral force on the central bubble is on the order of $10^{-6}\;\text{N}$, it is not strictly zero. This symmetry breaking phenomenon is more obvious in actual systems because minute differences in bubble size (only 1% deviation) can cause the central bubble to be subjected to a net attractive force pointing towards the larger bubble, leading to a tendency for the central bubble to deviate from the geometric center. Symmetry analysis also reveals the hierarchical structural characteristics of the system: outer bubbles constitute the first protective layer, and inner bubbles constitute the second protective layer. The effects of the two layers of bubbles on the central bubble involve both synergy (superposition of radiation pressure in the same direction) and competition (cancellation of radiation pressure in opposite directions). The balance between synergy and competition determines the final dynamic state of the central bubble.

The difference between square-wave and sinusoidal forcing arises from their distinct temporal and spectral characteristics. A finite-bandwidth square wave maintains an approximately constant tensile pressure over a larger fraction of the tensile half-cycle and contains multiple odd harmonics, whereas a sinusoid varies continuously and contains only the fundamental frequency. These differences can modify the timing and magnitude of bubble expansion and collapse [23], [24]. Nevertheless, the response must be interpreted together with the selected normalization criterion. Results obtained at equal peak pressure combine waveform effects with a difference in RMS forcing, while equal-RMS calculations provide a more appropriate basis for isolating the influence of waveform shape.

According to Eq. (6), a square wave can be expanded into a superposition of infinite odd harmonics, where the fundamental amplitude is $4P_{a}/\pi$, which is about 1.27 times the fundamental amplitude of a sine wave. More importantly, the square wave has theoretically infinite power density at the moment of positive and negative jumps. In actual calculation, due to series truncation N=25, the jump process is completed within about $10^{-8}\;\text{s}$. This sharp pressure jump produces a strong transient impact on the bubble. The instantaneous tensile force received by the bubble wall at the negative jump moment is much larger than the gentle stretching under sine wave driving, thus prompting the bubble to expand to a larger size in a shorter time. Numerical results confirm that under the same amplitude conditions, square wave driving increases the maximum radius of the central bubble by 46%, and the growth ratios for outer and inner bubbles are both above 15%. The high-frequency harmonic content of square waves [23], [24], [25] also has a significant impact on the bubble collapse stage. High-frequency components provide additional compression pulses when the bubble approaches the minimum radius, making collapse more thorough, and peak internal temperature and pressure increase markedly. Comparing Fig. 12, Fig. 14, the central bubble collapse temperature under square wave driving reaches 14200K, 109% higher than the 6800K under sinusoidal excitation. This elevated temperature represents stronger thermodynamic severity, yet it does not linearly correspond to higher oxidant yield. Air bubble simulations show an optimal temperature window for sonochemical radical generation; radical consumption via nitrogen oxidation dominates once temperatures exceed this optimum value [26], [27], [28], [29], [30], [31].

From a physical energy input perspective, square waves and sine waves have fundamentally different energy transfer mechanisms. Sine wave power input varies continuously and energy transfer proceeds gently; square waves maintain constant tensile pressure for half a cycle and constant compressive pressure for the other half, avoiding low-effort near-zero-crossing energy transfer stages. Quantitative analysis shows that the total energy absorbed by bubbles during the tensile half-cycle under square wave excitation is approximately 1.4 times that of sinusoidal driving. The rich odd-harmonic spectrum of square waves can simultaneously excite resonance modes of bubbles with different characteristic sizes, improving the overall energy absorption efficiency of the multi-bubble system. The hierarchical double-cube pressure field generated by mutual acoustic coupling of outer and inner bubbles leads to three distinct central bubble dynamic regimes: normal expansion, complete suppression, and delayed expansion. Complete suppression occurs when surrounding bubble positive radiation pressure fully counteracts external tensile acoustic pressure; delayed expansion arises when outer bubbles reach maximum volume and their radiation pressure switches from repulsive positive to tensile negative, superimposing with the external negative pressure to generate a strong net tensile force that drives delayed yet far more violent expansion and collapse.

The same numerical model produces qualitatively similar non-monotonic responses in the simplified three-bubble and five-bubble configurations. This cross-configuration consistency suggests that the competition between radiation-pressure shielding and collapse-assisted compression is not restricted to the double-cube arrangement. Nevertheless, the present evidence is numerical and limited to fixed-centre spherical bubbles; broader generalization requires validation using alternative spatial configurations and migration- or deformation-resolving models. Non-equilibrium vapour and temperature effects [26], [27], [28], [29], [30], [31], [32] jointly determine the extreme thermodynamic states generated during bubble collapse. Interactions quantified by secondary Bjerknes force [12], [13], [14], [15], [16], [17], [18] govern the spatial coupling and phase synchronization between all bubbles in the cluster. All spherical radial models adopted here carry inherent limitations associated with migration and deformation [33], [34], [35], [36], [37].

The present study distinguishes between three levels of evidence. First, reductions to the isolated- and coupled-bubble limits provide equation-level and numerical consistency checks. Second, qualitative agreement with previous coupled-bubble studies supports the predicted suppression mechanism. Third, the specific transition radii, regime boundaries, peak temperatures and force amplitudes reported for the double-cube configuration are original numerical results of the present model. These configuration-specific quantities are not treated as experimentally validated or universally applicable.

## 6 Conclusion

Based on the modified compressible Keller–Miksis multi-bubble coupling equation, this work establishes a fully coupled dynamic model for a hierarchically arranged double-cube 17-bubble system under finite-bandwidth square wave excitation. A complete 17×17 Cartesian pairwise distance matrix is constructed to accurately calculate inter-bubble acoustic radiation coupling, incorporating non-equilibrium interfacial evaporation/condensation and heat transfer instead of a simplified polytropic thermodynamic assumption. Systematic numerical simulations are carried out to analyze the radial oscillation characteristics, collapse extreme conditions, and secondary Bjerknes force distribution laws of outer, inner, and central bubbles, together with parametric sensitivity of geometric and acoustic driving variables. The core conclusions are summarized as follows:

1. Under the equal-peak-pressure reference condition, finite-bandwidth square-wave excitation produces a larger central-bubble expansion and a more intense calculated collapse than sinusoidal excitation. The central-bubble maximum radius increases from approximately 24μm to 35μm, while the calculated peak temperature increases from approximately 6800 K to 14200 K. Because the square wave has a higher RMS pressure at equal peak amplitude, these increases cannot be attributed solely to waveform shape. Equal-RMS calculations are therefore required to quantify the residual contribution of the extended tensile interval and odd-harmonic content.

2. The geometric scale ratio D/d between outer and inner cubes exerts a non-monotonic regulatory effect on central bubble expansion amplitude and collapse temperature. An optimal coupling state occurs at D/d≈2.0, where central bubble peak collapse temperature reaches 14200K. Smaller D/d strengthens radiation pressure shielding and induces complete central bubble suppression; larger D/d weakens inter-bubble coupling and the collapse amplification effect from surrounding bubble radiation pulses. Synchronous scaling of absolute cube dimensions reduces coupling strength monotonically and weakens inter-bubble interaction forces following an inverse-square distance law.

3. Driving frequency and acoustic pressure amplitude possess strong nonlinear control effects on central bubble dynamics. Increasing frequency from 20kHz to 35kHz shortens the available tensile expansion time, reducing the central bubble maximum radius by 49% and collapse temperature by 40%. Raising acoustic pressure amplitude from $1.05\times 10^{5}\;\text{Pa}$ to $1.5\times 10^{5}\;\text{Pa}$ strengthens net tensile loading, boosting peak collapse temperature by 348% and triggering the transition from weak oscillation, delayed expansion to fully developed normal expansion. A dimensionless regime map separating complete suppression, delayed expansion, and normal expansion is constructed via coupled $\Pi_{a}$-D/d parametric analysis.

4. Three characteristic dynamic modes of the central bubble are identified by adjusting surrounding bubble equilibrium size: normal expansion at small outer bubble radii, full suppression near $R_{o0}\approx 4.5\;\mu \text{m}$, and delayed expansion for $R_{o0}>6\;\mu \text{m}$. Delayed expansion produces drastically intensified collapse: peak inward wall velocity reaches 612m/s and peak temperature hits 28600K, exceeding both normal expansion states and equivalent isolated bubble cavitation severity. This amplification originates from synchronous superposition of surrounding collapse radiation pulses on the central bubble.

5. Cubic geometric symmetry leads to partial vector cancellation of secondary Bjerknes forces acting on the central bubble. Time-averaged net lateral force is near zero under perfectly uniform initial conditions, but nonlinear oscillation and numerical noise break symmetry to generate non-negligible instantaneous lateral loading. A mere 1% perturbation to a single outer bubble equilibrium radius creates a persistent net attractive lateral force pointing toward the enlarged bubble and weakens central bubble expansion amplitude. Inner bubbles exert stronger shielding and coupling effects than outer bubbles due to shorter central separation distances. The total secondary Bjerknes force magnitude decreases with rising driving frequency and grows nonlinearly with increasing acoustic pressure amplitude.

6. The spherical fixed-centre Keller–Miksis formulation adopted herein is quantitatively reliable only for subsonic wall Mach numbers $M_{w,\text{max}}<1$ and inter-bubble geometric separation criteria $\left(R_{i,\text{max}}+R_{j,\text{max}}\right)/D_{ij}<0.5$. Once these thresholds are exceeded, bubble translation and asymmetric deformation become prominent, requiring migration-resolving Zhang-type extended models incorporating dynamic drag and shape correction terms [33], [36], [37].

The proposed double-cube multi-bubble coupled model provides a novel theoretical framework for analyzing hierarchical clustered cavitation bubble interactions. The discovered central bubble suppression/delayed expansion mechanisms and square wave cavitation enhancement characteristics offer theoretical guidance for the precise tuning of ultrasonic cavitation in chemical processing, biomedical ultrasound, and precision cleaning engineering. Nevertheless, the current model retains several simplifying assumptions requiring further improvement in follow-up research: fixed bubble centroid positions, ideal spherical oscillation, infinite unbounded liquid domain, and neglect of solid boundary wall effects. Future work directions include: (i) Integrating bubble migration, asymmetric deformation, and boundary interaction terms into the governing equation system based on 2026 JFM Zhang-Xu extended migration models; (ii) Expanding simulation scale to large clusters containing over one hundred bubbles with diverse hierarchical geometric layouts; (iii) Conducting matching experimental characterization to validate numerical predictions of bubble radius evolution, collapse temperature, and inter-bubble interaction forces; (iv) Investigating alternative engineering-relevant cluster geometries such as spherical shell arrays and cylindrical layered bubble distributions to establish a generalized multi-bubble cavitation dynamic theoretical system, supporting refined control strategies for industrial acoustic cavitation technology.

## CRediT authorship contribution statement

**Wurihan Bao:** Writing – review & editing, Writing – original draft, Validation, Methodology, Funding acquisition, Formal analysis, Data curation, Conceptualization. **De-Xin Wang:** Writing – review & editing, Visualization, Validation, Supervision, Software. **Haiying Han:** Writing – review & editing, Writing – original draft, Visualization, Validation, Supervision, Software, Resources.

## Declaration of competing interest

The authors declare that they have no known competing financial interests or personal relationships that could have appeared to influence the work reported in this paper.

All authors confirm that there are no financial or non-financial conflicts of interest associated with this manuscript, including employment, consultancy, patents, grants, or other funding or commercial associations that might affect the impartiality of the research.
